# Does self-perceived HIV risk mediate the potential association between HIV-related symbolic stigma and sexual behaviour among young adult women in Cape Town, South Africa?

**DOI:** 10.1186/s12889-022-14862-7

**Published:** 2023-01-28

**Authors:** Takwanisa Machemedze

**Affiliations:** DataFirst, University of Cape Town, Cape Town, South Africa

**Keywords:** Risk perception, Symbolic stigma, Sexual behaviour, South Africa

## Abstract

**Background:**

Perception of risk is a central construct of models of health behaviour change as it is assumed to be an intermediate step before adoption of the related safer behaviour. In the context of HIV/AIDS, the literature suggests that psychosocial factors such as stigmatising attitudes related to stereotyping people who contract HIV may influence how people perceive their own risk of HIV infection. However, findings on the relationships between HIV-related stigma, HIV risk perception and sexual behaviour have been inconsistent. We investigated the potential mediating role of HIV risk perception on the link between HIV-related symbolic stigma and sexual behaviour.

**Methods:**

Data used in this study are a sub-sample of 384 young adult women, aged 17–25 years, who participated in the Cape Area Panel Study conducted in Cape Town, South Africa. Study participants were asked questions relating to their demographic details, their social and economic situation, and sexual and reproductive health behaviour. The outcome measure was a composite measure of sexual behaviour derived from whether the young adult women ever had sex before, previous number of sexual partners and condom use. The mediator variable was self-perceived risk of contracting HIV. The independent variable was HIV-related symbolic stigma attitudes. Mediation analysis within the structural equation modeling (SEM) framework was used to examine if participants who held elevated stigma attitudes perceived their risk of HIV infection to be low and as a result ended up engaging in unsafe sex.

**Results:**

Higher HIV-related symbolic stigma attitudes were associated with perception of reduced risk of contracting HIV (β = -0.248, *p* = 0.008, 95% CI = [-0.431, -0.066]) and perception of higher risk of contracting HIV was significantly associated with unsafe sex practices (β = 0.179, *p* = 0.038, 95% CI = [0.010, 0.348]). The indirect path was not significant (β = -0.044, *p* = 0.084, 95% CI = [-0.095, 0.006]), suggesting no mediation relationship.

**Conclusions:**

Stigmatising attitudes towards groups of people stereotyped as at risk of HIV infection was associated with perception of invulnerability to HIV, and the question on how this relationship affects risk sexual behaviour needs further investigation.

## Introduction

Sub-Saharan Africa remains the region most severely affected by HIV/AIDS over the course of the pandemic with over two thirds of the global number of people living with HIV/AIDS (PLWHA) in 2020 [1]. In 2013, sub-Saharan Africa accounted for 71% of people living with HIV/AIDS (PLWHA), 74% of AIDS-related deaths and about 1.5 million new HIV infections in the world [2]. The epidemic within the sub-Saharan Africa region also varies by country where South Africa contributes the greatest number of PLWHA [2]. Within South Africa, the distribution of the epidemic also varies by demographic attributes. A South African national survey conducted in 2012 showed that HIV prevalence was significantly higher among women (14.4%) compared to men (9.9%) [3]. The same survey showed that the biggest difference in HIV prevalence between male and female was among late teens (age group: 15–19, HIV prevalence: 0.7% vs 5.6%, i.e., 8 times higher) and young adults (age group: 20–24, HIV prevalence: 5.1% vs 17.4%, i.e., 3.4 times higher). In addition, nearly a quarter (24.1%) of all new HIV infections in 2012 were among young women aged 15–24 years. A similar pattern of HIV prevalence and incidence among young adults was also observed in a subsequent national survey [4]. As a result, young women are often identified as one of the key population vulnerable to HIV infection. In addition, the prevalence is high among Black African (15.0% in 2012, 16.6% in 2017) and Coloured (3.1% in 2012, 5.3% in 2017) compared to Indian/Asian (0.8% in both 2012 and 2017) and White (0.3% in 2012, 1.1% in 2017) population groups [3, 4].

Heterosexual sex is commonly assumed to be the main mode of HIV transmission in South Africa [3]. This is based on the observation that HIV prevalence and incidence is high among people who engage in heterosexual risk behaviours. For example, the high HIV prevalence and incidence among the young adult South Africans has been attributed to related heterosexual risky behaviours such as: early age at engagement in sexual activities, age difference with sexual partners, multiple sexual partners, and condomless sex [3, 4].

The international target as set out by the United Nations (UN) sustainable development goals (SDGs) is to end the HIV epidemic by 2030 [5]. The UN’s vision is to have zero new HIV infections, zero discrimination and zero AIDS-related deaths [6]. Subsequent South African national surveys show that eliminating new HIV infections remains a challenge especially among the 15–24 year age group [3, 4]. It is therefore important to understand why people engage in HIV-related risk behaviours as this can help to develop appropriate interventions to reduce new HIV infections and achieve the UN targets.

Theories of health behaviour provide frameworks that explain behaviours related to health risks. One of the commonly used frameworks is the Health Belief Model (HBM) [7] that was developed to explain why people do not always participate in prevention and screening programs for common diseases. The HBM places emphasis on an individual’s degree of fear of a health risk and their psychological barriers to taking action. The HBM implies that individuals make rational choices regarding their health based on their evaluation of the cost of the health risk against the cost of prevention. The resulting behaviour is hypothesised to be a function of an individual’s perceived susceptibility and perceived severity of a health condition, and the perceived benefits and barriers to behaviour change [8]. The HBM has been adapted to explain how one’s perception of the risk of contracting HIV influence their risky sexual behaviour practices. According to the HBM model, individuals who perceive themselves to be susceptible to the risk of HIV infection and consider the health risk of HIV/AIDS to be severe are likely to practice safer sex [9]. However, psychosocial factors such as stigmatising attitudes can be a barrier to HIV preventative behaviours. It is suggested that HIV/AIDS-related stigma attitudes based on moral judgement, blaming or stereotyping certain ‘out-groups’ of people for the spread of HIV creates perceptions of insusceptibility to HIV infection among the `in-groups’ and as a result, they do not practice safer sex [10]. Therefore, assessing the impact of HIV-related stigma on the relationships between perceived susceptibility to HIV and sexual risk taking is important for developing appropriate strategies to reduce the spread of HIV and theories for research.

HIV-related stigma from the general population can be grouped into two dimensions namely symbolic/social stigma and instrumental stigma. Symbolic stigma arises from moral judgements attached to people living with HIV/AIDS whereas instrumental stigma is a result of fear of the threat of HIV/AIDS and the desire to protect oneself from infection [11]. There is also another concept linked to HIV-related stigma based on people’s behavioural intentions to discriminate against people living with HIV [12]. A South African study shows that there was a general increase in the prevalence of the three dimensions of HIV-related stigma (i.e., symbolic, instrumental, behavioural intentions) in the mid-2000s [12]. However, a series of national surveys conducted between 2002 and 2017 observed a reduction in HIV-related stigma attitudes over the period [4]. Despite the observed reduction, one of the goals of the current South African national strategic plan is to combat HIV-related stigma [13]. This shows that HIV-related stigma is still a concern in South Africa and its potential role in undermining HIV prevention needs to be understood.

While the relationships between (a) HIV/AIDS-related stigma and risk perception and (b) HIV risk perception and sexual behaviour have been explored before, the results have not always been consistent. Findings appear to vary by context. For example, one South African study, conducted among young adults in Cape Town, found that high HIV/AIDS symbolic stigma was associated with reduced perceived risk of HIV infection [14]. On the contrary, another study based on a nationally representative South African survey found high stigmatising attitudes to be associated with perceptions of high risk of contracting HIV [15]. There are also studies finding conflicting results on the relationships between HIV risk perception and sexual behaviour. As expected based on the HBM, some studies found that individuals who perceive themselves at higher risk of infection with HIV were more likely to adopt safer sex practices [16, 17]. However, a study from Kenya found perceptions of elevated HIV risk to be associated with sexual risk taking [18]. Despite conflicting results in the association between perception of HIV risk and sexual risk taking, it is still relevant to evaluate how the HBM framework help to explain why some people fail to take precautionary measures against health risks.

Using data from the Cape Area Panel Study (CAPS), this study investigates the suggested mediation relationship, that is, whether HIV/AIDS-related symbolic stigma attitudes are associated with perception of low risk of infection with HIV, which then results in failure to take HIV prevention measures or engagement in risky sexual behaviours. The contribution of this study is to test the theorised mediation relationships simultaneously and evaluate the findings. Most studies test the individual hypotheses and connections are then implied based on results from different settings.

**Fig. 1:**
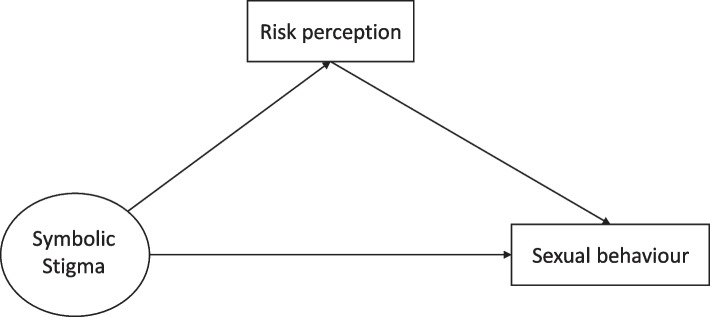
Conceptual mediation relationship

The conceptual framework of this study is shown in Fig. 1 and the hypotheses are as follows:

- *Hypothesis 1*: People who express elevated HIV/AIDS-related symbolic stigma attitudes perceive themselves at low risk of infection with HIV.
- *Hypothesis 2*: People who perceive themselves at low risk of infection with HIV engage in risky sexual behaviour.
- *Hypothesis 3*: HIV risk perception mediates the relationship between HIV/AIDS-symbolic stigma and sexual behaviour.

## Methods

### Study setting

We use data that was collected from the city of Cape Town, the largest metropolitan area in the Western Cape province of South Africa. According to the South African census conducted in 2001, the city of Cape Town population was 2 893 246, and this was about 6.5% of the population of South Africa [19]. The ethnic composition of the metropolitan Cape Town population was 31.7% African (black African), 48.1% Coloured (mixed race), 1.4% Indian or Asian, and 18.8% White. In comparison, the national population composition was 79.0% African, 8.9% Coloured, 2.5% Indian or Asian, and 9.6% White. Generally, the Western Cape province has the lowest HIV prevalence, which was about 5.1% in 2012 compared to the national prevalence of 12.6% [3]. The City of Cape Town (5.2%) is among the districts within the Western Cape that had HIV prevalence above the provincial average.

### Study design

The current study uses data from the Cape Area Panel Study (CAPS), a longitudinal study of young adults living in metropolitan Cape Town [20, 21]. The first wave of CAPS conducted in 2002 surveyed a sample of 4752 young adults aged between 14 and 22. The households from which the young adults came from were selected using a two-stage sampling design. The first stage selected a probability sample of census enumeration areas (EAs) from the 1996 South African census. The second stage randomly sampled households within each selected EA. In each recruited household, a household survey was administered to one adult who was knowledgeable about the household, and full-length youth questionnaire was administered separately to up to three young adults aged 14–22. The HIV-related stigma questions were first asked in wave 2 from a sub-sample of 1371 of the original respondents. The HIV-related stigma questions were asked again in wave 4 that was conducted in 2006, where the study successfully re-interviewed 3439 respondents. Creating a panel with complete data for the variables considered in this study yielded a smaller sample of 139 young adult Black and Coloured women participants across wave 2 and wave 4. We decided to use data from the fourth wave that yielded a bigger sample as described in sections to follow. Therefore, even though CAPS is a longitudinal study, the data for this analysis are cross-sectional and there is no follow-up.

### Study participants

UNAIDS [22] estimates that in 2019, 24% and 9% of new HV infections in the sub-Saharan Africa were among young women and men aged 15–24 years respectively. This is disproportionate compared to the other age groups. The current study focused on the sample of young adult Black (black African), and Coloured (mixed race) women aged between 17 and 25. This age group (17-25) was chosen because it covers the age range of the CAPS study in wave 4 and coincide with the age group disproportionately affected by new HIV infections as estimated by UNAIDS. Furthermore, young adult South African women in this age group (15–24 and 25–29) are identified to contract HIV at an early age compared to their male counterparts [3, 4]. In each CAPS wave of data collection, respondents were asked questions relating to their demographic details, their social and economic situation, and sexual and reproductive health behaviour. Young women who reported that they were HIV positive were excluded from the analysis.

### Measurement of variables

The current study is based on secondary analysis of data and the questions that were used to measure the various constructs were quoted as they appear in the questionnaire. The CAPS study documentation does not state how the questions were created but we observed that some of the questions on HIV risk perception, HIV/AIDS knowledge, and knowledge of someone with HIV/AIDS that were asked in the fourth wave conducted in 2003 where already introduced in the Demographic and Health Surveys (DHS) in 1995 [23] and 2001 [24]. We therefore assume that related questions on HIV risk perception, HIV/AIDS knowledge, and knowledge of someone with HIV/AIDS were adapted from standard international studies such as the Demographic and Health Surveys. The HIV-related stigma questions were invented for the CAPS study.

#### HIV/AIDS-related symbolic stigma

In the context of HIV/AIDS, symbolic stigma attitudes are based on moral judgement of people associated with HIV/AIDS. For example, in South Africa where HIV transmission is mainly through heterosexual sex, people with behaviours that are judged to be socially immoral such as promiscuity have been blamed for spreading HIV [25]. The current study assesses symbolic stigma attitudes from the general population by asking their opinion on the blameworthiness of PLWHA using the following questions: “Do you think HIV/AIDS is a punishment for sleeping around?”, “Do you think that many people who get HIV infected through sex have only themselves to blame?”. The possible responses to these questions were 1 (definitely yes), 2 (probably yes), 3 (probably no), 4 (definitely no) and 9 (Don’t know). In our analysis, the responses were reversed such that they are in increasing order of stigmatizing attitudes and the “don’t know” responses were treated as missing. HIV/AIDS-related symbolic stigma is treated as a latent variable as it is not directly measured but it is modelled as a two-item factor analysis in the model described in sections to follow.

#### Behavioural intentions

Previous studies operationalized a dimension of HIV-related stigma called behavioural intentions which is measured by asking respondents about how they would behave towards PLWHA under various hypothetical situations [12]. We use the measure of behavioural intentions to validate the symbolic stigma scale and this measure was assessed using the following questions: “Imagine that you find out that one of your friends is HIV infected. Would you still be friends with them?”, “If you knew that a shopkeeper had HIV/AIDS, would you buy fresh vegetables from him or her?”, and “Would you drink from the same bottle of water as an HIV infected friend?”. Responses to the behavioural intentions questions were similar to those of HIV/AIDS-related symbolic stigma described above. The behavioural intentions items are used to aid in testing the measurement model for symbolic stigma described above as it has fewer items than the minimum of three required.

#### HIV risk perception

HIV risk perception is one’s belief about their susceptibility to HIV infection. In the current study, HIV risk perceptions were assessed using the question, “Do you think you have no risk, a small risk, a moderate risk, or a great risk of getting the AIDS virus?” Response categories were: 1 (no risk), 2 (small risk), 3 (moderate risk), 4 (great risk), 5 (If volunteered: Is HIV positive), 8 (Refused) and 9 (Don’t know). Participants who reported that they were HIV positive were excluded from the analysis and those who “refused” or reported “don’t know” were treated as having missing data.

#### Sexual risk behaviour

Sexual risk behaviour is measured using a composite indicator for assessing “safe sexual behaviour among young people” as described by World Health Organization [26]. The composite indicator has six values described as follows:

- Respondents who have never had sex;
- Respondents who have had sex but not in the preceding 12 months;
- Respondents who had sex with only one partner in the preceding 12 months and who used a condom the last time;
- Respondents who had sex with only one partner in the preceding 12 months and who did not use a condom the last time;
- Respondents who had sex with more than one partner in the preceding 12 months and who used a condom the last time;
- Respondents who had sex with more than one partner in the preceding 12 months and who did not use a condom the last time.

The sexual risk behaviour composite measure is progressively riskier. It considers young adults who have had no partner, one partner and multiple partners over the preceding 12 months, and the frequency of condom use at the last sex among those people who have had only one partner or more than one.

#### HIV/AIDS knowledge

Health behaviour theories suggest that knowledge about a potential risk modifies one’s perception of the risk and behaviour towards the risk. As a result, several HIV/AIDS behavioral theories includes the concept of HIV/AIDS knowledge on the assumption that those who are better informed are more likely to practice safer sex [27]. As a result, the model adjusts for HIV/AIDS knowledge. We use a composite index for HIV/AIDS knowledge obtained by counting correct answers to the questions: “Do you think you can get HIV/AIDS by eating food prepared by someone with HIV/AIDS?”, “Do you think you can get HIV/AIDS by being coughed or sneezed on by someone who has HIV/AIDS?”, “Can people get HIV/AIDS because of witchcraft?” and “Is it possible for a healthy-looking person to have HIV?”. The responses were: “Yes”, “No”, “Maybe”, “Don’t know”. The answers considered to be correct were “No” for the first three questions and a “Yes” for the last question.

#### Education

Another measure related to HIV/AIDS knowledge is education attainment. It is argued that low education influences one’s perception about the risk of HIV infection and their approach towards preventive health behaviours [28]. Literature based on South African studies found that the level of education among young adults affects sexual risk behaviour in various ways. Some studies found no association between level of education and condom use [29] while other studies found higher level of education to be significantly associated with multiple sex partners [30]. As a result, the model adjusts for level of education. The level of education was measured by the number of completed years of schooling that was derived from the reported completed level of education. This is a continuous variable that measures the number of years that the participants have been in school.

#### Knowledge of someone with HIV/AIDS

Personal knowledge of someone with HIV/AIDS is also thought to influence risk perception, as individuals witness a person ill with AIDS and thus presumably adopt safer sexual behaviours [31]. The model adjusts for whether the participants personally knew someone with HIV/AIDS. This was assessed by asking the question, “Do you personally know anyone who has HIV/AIDS?” The responses were: “Yes”, “No”, “No response/refused” and “Don’t know”. The responses “No response/refused” and “Don’t know” were treated as missing resulting in a binary Yes/No variable.

### Data analysis

Descriptive statistics were performed using Stata 15 [32]. The mediation analysis was conducted within the structural equation modeling (SEM) framework and the analysis was performed in R using the *lavaan* package version 0.6–9 [33]. Since the model is based on ordinal outcome variables such as perception of risk, we used the diagonally weighted least squares (DWLS) estimator with robust mean- and variance-adjusted (WLSMV) test statistics [34].

The measurement model includes a latent variable for HIV/AIDS symbolic stigma. The symbolic stigma scale has only two items which makes it difficult to test the suitability of the scale. Our strategy for testing the symbolic stigma scale was to combine with the behavioural intentions scale described in the preceding sections. The suitability of the sample for factor analysis was assessed by the Bartlett’s Test of Sphericity (BToS) and the Kaiser–Meyer–Olkin (KMO) measure of sampling adequacy. A significant result for the BToS indicates sufficient covariance amongst the observed variables to justify the factor analysis and a KMO value of 0.60 or greater confirms suitability.

The fitness between the data and the hypothesised structural model was evaluated using the following indicators: (a) the root mean square error of approximation (RMSEA); (b) the standardized root mean square residual (SRMR); (c) the comparative fit index (CFI), and (d) the Tucker-Lewis index (TLI). A good model-data fit is indicated by SRMR < 0.08, RMSEA < 0.06, CFI > 0.95, and TLI > 0.95 [35].

## Results

### Descriptive statistics

We realised a final sample of 384 young adult women and Table 1 shows their characteristics. Participants were aged between 17–25 years, with an average of 10 years of schooling. Just over a third (37.5%) personally knew someone with HIV. Participants had good knowledge about HIV/AIDS as most of them (75.5%) successfully answered at least three of the four HIV/AIDS knowledge questions. Most of the participants perceived themselves at no risk (42.5%) or small risk (34.1%) of infection with HIV. About one in five (19.5%) of the participants had never had sex. Six out of ten (60.0%) of the participants had sex with one partner in the preceding 12 months, where about half of these (31.3%) used a condom the last time they had sex, and the other half (28.7%) did not use a condom at last sex.

**Table 1:** Sample characteristics: Cape Area Panel Study 2006

Characteristics	Range, Mean (SD), n (%)
**Age**	17—25, 21.0
**Years of schooling**	4—17, 10.5
**Personally know someone with HIV/AIDS**	144 (37.5%)
**HIV/AIDS knowledge (count and proportion of correct answers)**	
1. Do you think you can get HIV/AIDS by eating food prepared by someone with HIV/AIDS?	338 (88.0%)
2. Do you think you can get HIV/AIDS by being coughed or sneezed on by someone who has HIV/AIDS?	344 (89.6%)
3. Can people get HIV/AIDS because of witchcraft?	290 (75.5%)
4. Is it possible for a healthy-looking person to have HIV?	300 (78.1%)
**HIV/AIDS knowledge (Score of correct answers out of four questions)**	0—4, 3.3
**Respondent’s own assessment of risk of HIV infection**	
No risk	163 (42.5%)
Small risk	131 (34.1%)
Moderate risk	31 (8.1%)
Great risk	27 (7.0%)
Don’t know	32 (8.3%)
**Sexual risk behaviour (Composite indicator of increasing risk)**	
1—Ever had sex	75 (19.5%)
2—Had sex but not in the last 12 months	16 (4.2%)
3—Had sex with only one partner in the last 12 months and used a condom	120 (31.3%)
4—Had sex with only one partner in the last 12 months and did not use a condom	110 (28.7%)
5—Had sex with multiple partners in the last 12 months and used a condom	24 (6.3%)
6—Had sex with multiple partners in the last 12 months and did not use a condom	16 (4.2%)
Missing	23 (6.0%)
**N**	384

Table 2 shows results for the stigma scales used. The first two items measure HIV-related symbolic stigma, and the other three items measure behavioural intentions. If we consider grouping the responses into either “yes” (definitely/probably yes) or “no” (definitely/probably no), results in Table 2 shows that about four out of ten (39.8%) of the participants think that HIV/AIDS is a punishment for sleeping around and just over half (52.1%) think that people who are HIV positive only have themselves to blame.

**Table 2:** Indications of HIV-related symbolic stigma and behavioural intentions

Questions asked	Definitely yes	Probably yes	Probably no	Definitively no	Don’t know
1. Is HIV/AIDS punishment for sleeping around? (*symbolic stigma*)	78 (20.3%)	75 (19.5%)	70 (18.2%)	130 (33.9%)	31 (8.1%)
2. Do many HIV + people have only themselves to blame? (*symbolic stigma*)	111 (28.9%)	79 (20.6%)	63 (16.4%)	110 (28.7%)	21 (5.5%)
3. Imagine that you find out that one of your friends is HIV infected. Would you still be friends with them? (*behavioural intentions*)	306 (79.7%)	16 (4.20%)	1 (0.3%)	59 (15.4%)	2 (0.5%)
4. If you knew that a shopkeeper had HIV/AIDS, would you buy fresh vegetables from him or her? (*behavioural intentions*)	241 (62.8%)	29 (7.6%)	18 (4.7%)	88 (22.9%)	8 (2.1%)
5. Would you drink from the same bottle of water as an HIV infected friend? (*behavioural intentions*)	190 (49.5%)	64 (16.7%)	35 (9.1%)	76 (19.8%)	19 (5.0%)

### Mediation analysis

#### Measurement model

We tested the suitability of the HIV-related symbolic stigma scale for factor analysis. The Bartlett’s test for both the symbolic stigma attitudes and behavioural intentions was significant (χ^2^ = 242.78, *df* = 10, *p* < 0.05), indicating that correlations exist among some of the items. The Kaiser–Meyer–Olkin (KMO) measure was 0.55, indicating that the data were reasonably appropriate for this analysis. Exploratory factor analysis was performed and loadings less than 0.30 were excluded. The EFA yielded two factors that confirm the two latent variables, i.e., behavioural intentions and symbolic stigma attitudes. The standardized Cronbach’s alpha coefficient of reliability for the behavioural intentions’ items (α = 0.58) was satisfactory, and very good for the symbolic stigma items (α = 0.75).

#### Structural model

**Fig. 2:**
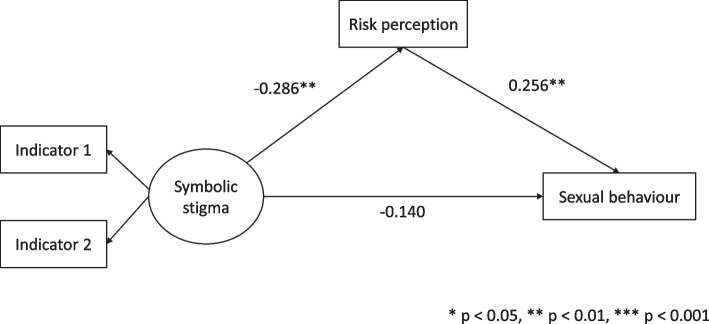
Results for the base mediation model

To test the hypotheses stated above, mediation analysis within a structural equation modeling framework was used. First, we tested the mediation role of HIV risk perception on the relationship between HIV/AIDS-related symbolic stigma attitudes and sexual behaviour without adjusting for other variables. The standardized fit indices indicated that the model with no covariates was appropriate: the RMSEA was 0.010, the SRMR was 0.015, the CFI was 1.000, and the TLI was 1.000. The standardized estimates for the structural model are shown in Fig. 2 and the relationships between the variables were examined.

The direct relationship between symbolic stigma and sexual behaviour was not significant (β = -0.140, *p* = 0.297, 95% CI = [-0.404, 0.123]). The results indicated that HIV/AIDS-related symbolic stigma had a significant negative association with risk perception (β = -0.286, *p* = 0.002, 95% CI = [-0.470, -0.103]), suggesting that people who express symbolic stigma attitudes perceive themselves at low risk of infection with HIV. As a result, the hypothesis that people who express elevated HIV/AIDS-related symbolic stigma attitudes perceive themselves at low risk of infection with HIV was supported by the data. Furthermore, perception of risk had a significant positive relationship with sexual risk behaviour (β = 0.256, *p* = 0.005, 95% CI = [0.076, 0.436]). Therefore, the hypothesis that people who perceive themselves at low risk of infection with HIV engage in risky sexual behaviour was not supported by the data. The indirect path linking symbolic stigma and sexual behaviour, estimated by the product of the respective coefficients was significant (β = -0.073, *p* = 0.031, 95% CI = [-0.140, -0.007]). This suggest that perception of HIV risk is a significant mediator in the relationship between symbolic stigma and sexual behaviour. While the mediated relationship is significant, the direction of the hypothesised association was not supported.

**Fig. 3:**
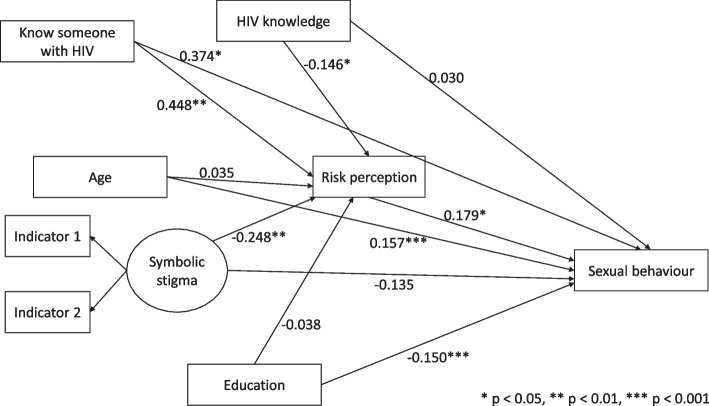
Results for the adjusted mediation model

We further explored the model by adding variables which have previously been observed to predict both risk perception and sexual behaviour and these are: age, education (years of schooling), HIV/AIDS knowledge and knowledge of someone with HIV/AIDS. The standardized fit indices for the adjusted model shown in Fig. 3 indicated that the model was appropriate: the RMSEA was 0.050, the SRMR was 0.017, the CFI was 0.984, and the TLI was 0.990.

Results from the second model in Fig. 3 indicated that the direct relationship between symbolic stigma and sexual behaviour was not significant (β = -0.135, *p* = 0.295, 95% CI = [-0.387, 0.117]). Symbolic stigma attitudes were associated with perception of reduced risk of contracting HIV (β = -0.248, *p* = 0.008, 95% CI = [-0.431, -0.066]), suggesting that people who expressed symbolic stigma attitudes perceived themselves at low risk of infection with HIV. Again, the hypothesis that people who express elevated HIV/AIDS-related symbolic stigma attitudes perceive themselves at low risk of infection with HIV was supported. Furthermore, perception of risk was demonstrated to be positively associated with sexual risk behaviour (β = 0.179, *p* = 0.038, 95% CI = [0.010, 0.348]). Thus, the hypothesis that people who perceive themselves at low risk of infection with HIV engage in risky sexual behaviour was not supported. The indirect path was not significant suggesting that perception of risk is not a significant mediator in the relationship between HIV/AIDS-related symbolic stigma and sexual behaviour (β = -0.044, *p* = 0.084, 95% CI = [-0.095, 0.006]). As a result, the hypothesis that self-perceived risk of HIV infection mediates the relationship between HIV/AIDS-symbolic stigma and sexual behaviour was not supported by the data.

Among the covariates included in Fig. 3, knowledge of someone with HIV/AIDS was associated with perception of higher risk of contracting HIV (β = 0.448, *p* = 0.001, 95% CI = [0.175, 0.721]) and better knowledge about HIV/AIDS was associated with perception of reduced risk of HIV infection (β = -0.146, *p* = 0.043, 95% CI = [-0.289, -0.004]). More years of schooling (β = -0.150, *p* = 0.000, 95% CI = [-0.233, -0.068]) was associated with reduced sexual risk behaviour. Increasing age (β = 0.157, *p* = 0.000, 95% CI = [0.085, 0.229]) and knowledge of someone with HIV/AIDS (β = 0.374, *p* = 0.033, 95% CI = [0.030, 0.718]) were both associated with engagement in risky sexual behaviour.

## Discussion

The current study seeks to contribute to the literature by investigating the link between HIV/AIDS-related symbolic stigma and sexual risk behaviour through the mediating effect of self-perceived risk of contracting HIV. We used data on young adult women collected from the Cape Area Panel Study to establish three findings. First, our results support the hypothesis that elevated HIV/AIDS-related symbolic stigmatising attitudes were associated with perception of reduced risk of contracting HIV. Second, perception of high risk of contracting HIV was associated with risky sexual behaviour, contrary to health behaviour theories. Third, by implication of the second result, the hypothesised mediation role of the perception of HIV risk on the relationship between HIV/AIDS-related symbolic stigma attitudes and risky sexual behaviour was not supported.

The significant relationship between HIV/AIDS-related symbolic stigma attitudes and perceived risk of contracting HIV is consistent with several studies [10, 14, 36]. HIV/AIDS—symbolic stigma attitudes are linked to stereotype beliefs about people who are likely to contract HIV. The AIDS risk reduction model (ARRM), which is one of the health behaviour theories, hypothesise that the process of behaviour change involves an individual to first identify and label a behaviour as risky before making the commitment to change it [37]. It is in this context where links are made to suggest that individuals who hold stereotypical ideas about people who are likely to contract HIV will not identify themselves to be at risk of HIV infection. This finding adds to the body of literature on the potential effects of HIV/AIDS-related symbolic stigma on risk perception. HIV-risk reduction interventions targeting risk perception might be guided by taking into consideration the role of HIV/AIDS-related symbolic stigma attitudes.

Our study found a significant positive relationship between HIV risk perception and sexual risk behaviour but this is not consistent with what is hypothesised in health behaviour theories [7, 37]. Risk perception is a central construct of models of health behaviour change as it is assumed that individuals who perceive their health to be at risk are more likely to adopt the related safer behaviour. We expected our results to show an association between perception of high risk of contracting HIV and the practice of safer sex. One possible explanation for our results could be that we are using cross-sectional data to predict variables that may have temporal precedence. Current perceptions of being at risk are expected to influence future behaviour. By using cross-sectional data, we are taking current reported risk perception to predict current or previous sexual behaviours. It is therefore possible that those who have already engaged in risky sexual behaviour perceive themselves at risk of contracting HIV. This finding is also consistent with other studies [18]. Instead of predicting previous or current sexual behaviour, Riley and Baah-Odoom [10] predicted intended sexual behaviour and found perceived vulnerability to be associated with intention to practice safe sex.

It is also important to note that the relationship between self-perceived risk of HIV infection and sexual risk behaviour has not always been clear. One South African study of young women observed that there was no difference in the reporting of risk perception between participants who had tested HIV-negative and those who tested HIV-positive [14]. The finding that there was no difference in self-perceived risk between those who had engaged in risk sexual behaviour and those who had not engaged in risk sexual behaviour is thought to reflect inaccuracies in self-evaluation of one’s vulnerability to HIV. It is also important to note that the question on risk perception did not specify a timeframe, so it was not clear whether participants were thinking of their immediate risk or their lifetime risk of acquiring HIV. A study of young people in Ghana argued that societal norms and practices can undermine young people’s construction of HIV risk and safer sex practices [38]. As a result, social norms can be a central determinant of sexual behaviour practices in addition to self-perceived HIV risk. This calls for more awareness messaging around sexual practices and the risk of HIV infection.

Overall, this study could not establish the hypothesised mediation role of self-perceived HIV risk on the relationship between HIV/AIDS-related symbolic stigma and sexual behaviour. This finding is consistent with another study [10] that found no association between current perception of risk and current or past sexual behaviour. The same study further observed that perceptions of HIV risk were associated with intended sexual behaviour as opposed to current or past sexual behaviours.

The contribution of the current study was to test the hypothesized mediation relationships simultaneously and evaluate the overall relationships. Several studies have independently tested the bivariate relationships between HIV-symbolic stigma and risk perception [14, 15], and HIV risk perception and sexual behaviour [16–18]. Finding from some of these studies can be interpreted to imply the mediation hypothesis tested in this study [10]. The current study is among a few other studies that went a step further and tested these relationships simultaneously using data from the same subjects [10, 36].

## Limitations

Our study used cross-sectional data and it is therefore not possible to make causal inferences. We investigated whether self-perceived vulnerability to HIV influences one’s sexual behaviour practices. As a result of the cross-sectional nature of the data, our model is using current self-perceived risk to predict most likely previous or current sexual behaviour as opposed to future behaviour. Using carefully designed longitudinal data can help to test this relationship taking into consideration the temporal precedence of occurrence of the behaviours. Another alternative is to ask participants in a cross-sectional study about their intended sexual behaviour as was done elsewhere [10].

Data used in this study were collected in 2006, and much of the ongoing anti-stigma campaign and awareness education may have changed stigma perceptions as well as risky sexual behaviours if this were to be replicated currently.

There are also several limitations in our measures. Our study used two items to measure symbolic stigma attitudes, and this may not fully capture the concept that is being measured. Similarly, we also used a single item to measure perception of risk. While these measures can be improved, other scholars have used the same data and their limitations to establish new insights [14]. All measures used in the study are self-reported by participants and these may be affected by social desirability. Our study used data based on a sample of young adults from Cape Town, South Africa. This limits the generalisability of our findings to other contexts. Another limitation is that we excluded young women who volunteered to report that they were living with HIV as an optional answer for the HIV risk perception question, but HIV status was never assessed directly. It is possible that some respondents were already living with HIV but did not volunteer their HIV status and their responses were likely to distort some of the tested relationships. Despite these limitations, our study produced plausible results that are consistent with other studies.

## Conclusions

The study established that HIV/AIDS-related symbolic stigma attitudes potentially undermine people’s self-evaluation of their vulnerability to HIV. This is troubling to know as risk misjudgement may also undermine caution in sexual risk taking. We could not establish the overall theorised mediated relationships between HIV-related symbolic stigma and sexual behaviour. Using longitudinal data may help to establish whether perception of the risk of contracting HIV mediate the relationship between HIV/AIDS-related stigma and sexual behaviour. It is also important to establish other factors that play a role in the relationships between HIV/AIDS-related stigma and prevention practices.

## Data Availability

The data that support the findings of this study are available in the DataFirst repository, https://www.datafirst.uct.ac.za/dataportal/index.php/catalog/266.

## Abbreviations

AIDS: Acquired immunodeficiency syndrome
BToS: Bartlett’s Test of Sphericity
CAPS: Cape Area Panel Study
CFI: Comparative fit index
DHS: Demographic and Health Survey
DWLS: Diagonally weighted least squares
EA: Enumeration area
HBM: Health belief model
HIV: Human immunodeficiency virus
KMO: Kaiser-Meyer-Olkin
PLWHA: People living with HIV/AIDS
RMSEA: Root mean square error of approximation
SDG: Sustainable development goals
SEM: Structural equation modeling
SRMR: Standardized root mean square residual
TLI: Tucker-Lewis index
UN: United Nations
WHO: World Health Organization
WLSMV: Weighted least squares mean- and variance-adjusted
